# Supporting Adolescents with Mental Health Problems in Secondary Education: Feasibility of a Supported Education Intervention

**DOI:** 10.3390/ijerph19116754

**Published:** 2022-05-31

**Authors:** Lindy Beukema, Jacomijn Hofstra, Sijmen A. Reijneveld, Andrea F. de Winter, E. L. Korevaar

**Affiliations:** 1 Department of Health Sciences, University Medical Center Groningen, University of Groningen, 9700 RB Groningen, The Netherlands; s.a.reijneveld@umcg.nl (S.A.R.); a.f.de.winter@umcg.nl (A.F.d.W.); 2 Research and Innovation Centre for Rehabilitation, Hanze University of Applied Sciences, 9747 AS Groningen, The Netherlands; j.hofstra@pl.hanze.nl (J.H.); e.l.korevaar@pl.hanze.nl (E.L.K.)

**Keywords:** mental health, adolescents, secondary school, participation, feasibility, intervention

## Abstract

Mental health problems in adolescence can have a profound influence on school functioning, educational attainment and thus future societal participation. Supported education (SEd) is a potentially useful method for educational professionals to help adolescents with mental health problems in secondary school improve their functioning by stimulating collaboration, ownership, and participation. In this study, we examined the feasibility of SEd in secondary education by examining its acceptability, implementation, and preliminary effectiveness. We performed a mixed-methods study using quantitative data (questionnaires) and qualitative data (interviews) from educational professionals (EP) and adolescents, aged 13–17, about their experiences with a SEd intervention. Regarding the acceptability of the intervention, three main themes emerged: (a) structure, (b) autonomy, and (c) applicability of the intervention. Themes regarding the implementation were: (a) lack of time, (b) personal attitude, (c) mastery, and (d) complexity of the school environment. The findings show that, for those that followed the intervention, SEd is a promising approach to support adolescents with mental health problems to improve their functioning and participation in school. Further research is needed on the effectiveness of the intervention.

## 1. Introduction

Worldwide, mental health problems occur frequently in adolescence [1,2,3,4] and can have a profound influence on school functioning [5]. Most long-term school absence and school drop-out in secondary school are related to mental health problems [6]. In addition, adolescents with mental health problems are more likely to finish school with a low educational level [7]. Hereby, mental health problems can have negative consequences for adolescents’ educational, social, work, and mental outcomes later in life [8,9].

Studies have shown that schools can be a source of support for adolescents with mental health problems and can prevent their school absence and drop-out [10,11,12,13]. However, whereas most mainstream schools provide support services for issues such as learning disabilities, they do not routinely do so for adolescents with mental health problems. The available mental health interventions for adolescents mostly focus on specific psychiatric disorders [14,15] on general awareness [10] and/or on mental health literacy and stigma [16]. Evidence is much scarcer on the use and effectiveness of interventions and support strategies that help adolescents with mental health problems to stay at secondary school and attain their diploma [10,17,18].

Supported education (SEd) [19] has been shown to be promising in post-secondary education to help young people with mental health problems in realizing educational goals [20] and could similarly help adolescents with mental health problems in secondary education, being a potentially useful tool for educational professionals working with them. SEd is originally based on the psychiatric rehabilitation approach of Boston University [21,22]. This approach assumes that problems resulting from a mental health issue can be compensated for by services, skills training, and support. SEd has been shown to empower and improve school efficacy and to lead to better, more specific goal-setting by students as well as greater educational engagement [23].

SEd is a tailored intervention based on the personal needs of the individual and includes the following principles: (a) focus on the student role, (b) collaboration, and (c) ownership. Focus on the student role refers to the fact that SEd is not therapy or mental health counseling but focusses on the problems in school functioning related to the students’ mental health. Goalsetting in SEd therefore also aims at practical solutions regarding these problems. This also means that the providers of the intervention do not need in-depth knowledge regarding mental health disorders. Collaboration means that students are actively involved in every step of SEd and are seen as a partner: goals are determined through shared decision making. Ownership means that throughout the support process, the students’ wishes are respected, and they are allowed to control and fully participate in determining the criteria for success and satisfaction and evaluate their own progress in terms of their goals. These principles have in common that they aim to stimulate competencies that help adolescents take charge of their own problems and their solutions and to be able to participate in school to their full potential.

SEd consists of three parts: choose, get, and keep (for more details on the SEd intervention, see Table 1). In the current study, we focus on the keep part of the intervention. This part focusses on how to support those with mental health problems to remain at school and how to increase their school success and satisfaction through the development of skills and better use of resources that are important in an educational setting [19]. The focus of the choose and get phases is on adolescents with mental health problems who dropped out of school because of their problems but want to return to school and need help with choosing and obtaining an educational setting of their own preference. It is important to note that SEd is not a mental health intervention but can exist next to and in addition to the youth mental health care system. Fortunately, the topic of promoting youth mental health is gaining more and more attention, but there is still a need for a more integrated youth mental health care system [24].

Before investigating the effectiveness of the SEd keep intervention, we should first address its feasibility for the new target setting of secondary education. Therefore, the aim of the current study is to evaluate whether the SEd keep intervention (further: SEd Intervention) is a feasible intervention for educational professionals and adolescents in secondary education. In order to evaluate the feasibility, we assessed the (1) the preliminary effectiveness, which regards whether the intervention shows promise of being successful with the intended population; (2) the acceptability, which regards how the intended individual recipients—both targeted individuals and those involved in implementation—react to the intervention; and (3) the implementation of the intervention, which concerns the extent, likelihood, and manner in which an intervention can be fully implemented as planned and proposed, in accordance with the feasibility aspects described by Bowen et al. [25].

## 2. Materials and Methods

We used a mixed-methods approach combining qualitative and quantitative methods to evaluate the feasibility of the intervention. First, educational professionals (EP) working in secondary education were trained in the SEd intervention. Second, these EP recruited adolescents from their schools and implemented the intervention in their counseling sessions, which was evaluated using questionnaires. Third, EP and adolescents were interviewed about the intervention.

### 2.1. Participants

**Figure 1:**
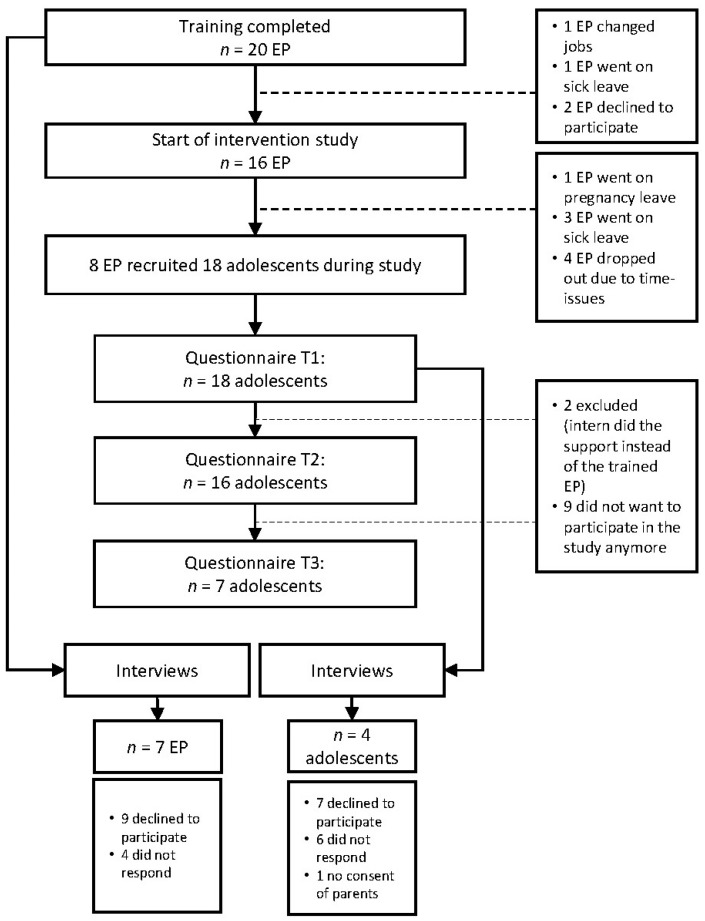
Participant Flow Chart.

Twenty EP from ten secondary schools in the Netherlands participated in the study between March 2017 and July 2019. For an overview of the entire participant flow, see Figure 1. Of the 20 trained EP, eight eventually participated in the study by recruiting adolescents and administering the intervention. The not-participating EP reported that they were unable to perform the intervention due to sick leave (e.g., burn-out), lack of time, and/or change of jobs. Of the 18 adolescents (56% female, mean age of 14 years), 11 dropped out from T1 to T3. EP reported the following reasons for the adolescents’ drop-out: not wanting to receive support in school anymore, lack of time of the EP, or more pressing issues, such as therapy outside of school, physical illness, or focus on schoolwork, due to which the support was initially put on hold but did not restart in the time frame of our study.

The EP were all working in the participating schools as counselors, school psychologists, or pedagogists. The inclusion criterium for EP was whether their work involved individual counseling of adolescents in school. Inclusion criteria for adolescents were: being in secondary education and having any type of emotional or behavioral problem that interfered with the adolescents’ school functioning. They were excluded in case of learning problems due to intellectual disability.

### 2.2. Procedure

#### 2.2.1. Training in the Supported Education Intervention

The EP were trained in the intervention by an experienced SEd trainer. The training took place in six sessions (three full days and three mornings) spread over four months. In the first three sessions, the theory behind the intervention was explained through presentations and practical assignments (e.g., role plays). The EP were asked to practice the intervention with an adolescent in-between training sessions. The last three sessions were used to discuss questions and issues regarding specific cases with which the EP were practicing the intervention. For more details on the training see Appendix A.

The intervention was performed by the EP at school. EP were told to have sessions with one adolescent for 12 weeks. The duration of one session could range between 45–55 min (the duration of one class).

#### 2.2.2. Data Collection: Questionnaire

The EP informed the adolescents that received counseling from them at school (and one of the parents if the pupil was younger than 16) about the study. Then, the EP, the adolescent, and, if necessary, a parent signed an informed consent form. To examine the preliminary effectiveness, the adolescent filled in three questionnaires: (a) before the start of the intervention (T1) and (b) six and (c) twelve weeks after the start (i.e., approximately half-way, T2) as well as at the end (T3).

#### 2.2.3. Data Collection: Interviews

We developed two interview guides to examine acceptability and implementation: one for the EP and one for the adolescents. Both were pilot-tested with one participant, which did not lead to changes.

After the adolescents filled in the T2 questionnaire, they and the twenty trained EP were invited for an interview. For the interview, we again asked EP and adolescents (and if necessary, a parent) to sign an informed consent form. In the end, 7 EP and 4 adolescents agreed to an interview. All participants and, if necessary, a parent, signed an informed consent form. The interviews with the EP were conducted by a research assistant (RD) without a previous established relationship with the EP and the adolescents’ interviews by the first author (LB), who did not have any relationship to the adolescents. All interviews were recorded with an audio recorder. The interviews with the EP were transcribed by R.D. and the adolescents’ interviews by L.B. For further information on coding and analyzing, see Data Analysis.

Interview results of adolescents were not shared with the EP or vice versa. Before the interviews, adolescents were assured of the anonymity of their answers towards the EP.

### 2.3. Measures

#### 2.3.1. Questionnaire

The main measures in the questionnaire regarded school functioning, psychosocial problems, and level of self-efficacy. The secondary outcomes were goal attainment, improved understanding, and improved confidence.

School functioning of the adolescent was measured using the 6-item self-report school functioning subscale of the Dutch version of the PedsQL (ages 13–18 years) [26] and was assessed at T0, T2, and T3. The psychometric properties were found to be adequate in a Dutch adolescent population (alpha ranged between 0.62 and 0.85) [27]. Sample items include: “How often did you have trouble with paying attention in class in the last month?” and “How often did you have trouble with skipping school in the last month?”. The items could be answered on a 5-point scale ranging from never, rarely, sometimes, often, or almost always a problem. A total score on the subscale was calculated by dividing the sum of the item scores by the number of items and ranges from 0–100. A higher score indicates better functioning.

Psychosocial problems of the adolescent were measured with the 25-item Dutch self-report version of the Strengths and Difficulties Questionnaire (SDQ) [28,29,30] with acceptable internal consistency (alpha = 0.78, test-retest stability (ICC = 0.87) and good concurrent validity [29] and were assessed at T1 and T3. Sample items include: “I worry a lot”, “I am easily distracted, I find it difficult to concentrate”, and “Other children or young people pick on me or bully me”. Items were answered on a scale from not true (1), somewhat true (2), and certainly true (3). The item scores were recoded and summed to calculate the Total Difficulties Score (see www.sdqinfo.org, accessed on 30 April 2020) for the complete scoring instruction). The Total Difficulties Score (TDS) ranges from 0–40, with a higher score indicating more problems. The Dutch norm in adolescents is a TDS below 11 [31].

Self-efficacy was assessed, with the Dutch version of the 10-item General Self-Efficacy Scale (GSES) [32] to measure the adolescents’ perceived self-efficacy in regard to coping with daily and stressful events, at T1 and T3. The instrument is considered to be valid and reliable (alpha = 0.84) [33,34]. Sample items include: “I can always manage to solve difficult problems if I try hard enough”, and “I can usually handle whatever comes my way”. The items are measured on a 4-point scale from completely disagree to completely agree. The total score is defined as the sum of all items and ranges from 10–40, with a higher score indicating more self-efficacy.

Goal attainment was assessed with The Goal Attainment Scale (GAS) [35,36], an individualized and standardized evaluation method, which we used to assess the extent to which the adolescents’ goals (determined at baseline) are attained. The GAS was assessed at T2 and T3 by asking “To what extent did you achieve goal X” and was measured on a 5-point answer scale ranging from “worse than expected”, “less than expected”, “expected”, “more than expected”, to “best expected”. For the purpose of this study, answers were recoded into the categories: not obtained, partly obtained, and completely obtained.

Improved understanding was assessed by 1 item asking the adolescents to indicate how much they had learned from the support they received. The question included examples of what they could have learned, such as a better understanding of the problems and/or knowing how to handle difficult situations better.

Improved confidence was similarly assessed by 1 item asking the adolescents to indicate how much their feelings had changed positively because of the intervention on a scale from 1 to 10. The specific question was: “Please indicate how much your feelings have changed due to the support you have received”. The question also included examples of what feelings might have been improved, such as improved self-confidence, worrying less, and/or feeling less hopeless.

Background characteristics included adolescent characteristics, such as age, gender, and educational level, and EP characteristics, including age, gender, and years of counseling experience. Educational level was categorized into lower secondary education level (lower vocational and lower secondary) and intermediate/higher secondary education level. Ethnicity was defined as non-Dutch if the adolescent or at least one of his/her biological parents was born outside the Netherlands.

#### 2.3.2. Interviews

The interviews with EP and adolescents addressed the acceptability and implementation of the intervention. Acceptability is defined as how the intended individual recipients—both targeted individuals and those involved in implementation—react to the intervention. Implementation is defined as concerning the extent, likelihood, and manner in which an intervention can be fully implemented as planned and proposed [25].

The interview guide for the trained EP focused on how they perceived the acceptability of the intervention for themselves and for the adolescents. The questions on implementation were based on the MIDI implementation determinants instrument [37].

The interview guide for the adolescents focused on their perspectives and experiences regarding acceptability of the SEd intervention. We used three main questions: (1) “What did you do during the counseling sessions and what did you think about that?”, (2) “What did you learn from the counseling sessions?”, and (3) “How do you feel about the counselor?”. These questions were an extension of those asked in the questionnaire.

Interviews lasted between 30 min and one hour, with the variation being mostly due to the length of the answers of the participants.

### 2.4. Data Analysis

#### 2.4.1. Questionnaire

First, we described the background of the sample. Then, to assess the preliminary effectiveness, we described the outcomes on the variables improved confidence, improved understanding, and the GAS in means and percentages. We used SPSS (version 26, IBM, Armonk, NY, USA) for the analysis of the questionnaire data.

#### 2.4.2. Interviews

We completed a thematic analysis [38,39] of the interviews using Atlas.ti computer software (version 8; Atlas.ti Scientific Software Development GmbH, Mannheim, Germany). Codes were derived from the data rather than according to a pre-existing theoretical framework. The first author (L.B.) and the second author (J.H.) coded two transcripts separately and discussed and compared their results until consensus was reached. The first author then coded the other interviews. The codes and themes were revisited and refined after each new transcript was coded. Since we used a structured interview format, we started with defining main themes based on the interview questions. Codes were then assigned and gradually organized into these main themes. Finally, the themes and codes were discussed with all the co-authors and adjusted before finalizing and reporting the analysis.

## 3. Results

### 3.1. Participant Characteristics

Table 2 provides information on the background characteristics of the EP and adolescents. The majority of the EP were female and had an average of 14 years of experience in counseling adolescents.

Of the 18 adolescents at T1, most received support because of their internalizing problems, such as depressive thoughts, feeling panic or being overwhelmed by schoolwork, or perfectionism. The second most mentioned type of problems was of externalizing nature and related to behavior towards and relationship with teachers or peers. Other often mentioned reasons for receiving support were motivation and attitude regarding homework and school, planning and organization problems, and absenteeism. Regarding four adolescents, the home situation also played a role.

### 3.2. Preliminary Effectiveness

Table 3 shows the means and percentages of the main outcome measures based on the small number of participants left at T3 (*n* = 7). Mean SDQ was 15 (SD 4.0) at T1 and 11 (SD 3.5) at T3, indicating a slight decline in mean SDQ scores. In regard to the GAS, four of the seven participants at T3 reported that they obtained at least one of their goals during the intervention. The other three participants reported they partly obtained one or more goals.

### 3.3. Acceptability of the Intervention

#### 3.3.1. Educational Professionals (EP)

Three main themes regarding the acceptability of the intervention emerged: (a) structure, (b) autonomy, and (c) applicability. The themes are summarized with supporting quotes in Table 4. With regard to the structure of the intervention, all EP agreed that the main strength of the intervention is its schematic approach and the supportive tools: every step includes writing down the problems, answers, or solutions in diagrams or tables. This supports the counselor as well as the adolescent. However, the EP also had some points of improvement. They suggested to create a more concise manual that was more compact in the number of papers and the lay-out of the pages.

The second theme that emerged as a positive aspect was autonomy. The EP felt that the intervention forced them to not immediately solve the adolescent’s problem but to let the adolescents think of solutions themselves. In addition, EP noted that the intervention has the potential to stimulate more self-awareness, independence, and autonomy in the adolescents.

The third theme that emerged regarded the applicability of the intervention, on which opinions differed. Some EP suggested that the intervention is not sufficiently applicable to all adolescents, as it depends on their level of self-reflective abilities, level of self-awareness, motivation, or involvement of parents. Other EP found the intervention to be applicable to every adolescent and described it as depending on the skills of the counselor to adapt the intervention to the level of the adolescent.

#### 3.3.2. Adolescents

Overall, the adolescents were satisfied with the counseling they received. Four themes emerged from the interviews: (a) non-judgmental listening, (b) shared decision making, (c) structure, and (d) visualization. The first theme regarded non-judgmental listening. The adolescents reported that they appreciated to have someone to talk to about the problems they face without having the feeling of being judged. They described that their teachers often just get angry but that the counselors listened and made them feel taken seriously. The second theme that emerged was shared decision making. The adolescents described how they together with the EP thought about and tried different solutions for their concentration problems or anger problems and how they tried to connect the solution to a skill they could learn (e.g., social, emotion-regulation, or study skills) or a resource they could employ for assistance (e.g., study buddy, teacher, stress ball). A third theme that emerged was structure. The adolescents appreciated analyzing the situations in which something went wrong, step by step, together with the EP. The fourth theme that emerged was visualization. More specifically, many adolescents described how either the counselor or they jointly wrote things down on a whiteboard or paper and how this helped them to better understand their problems and potential solutions.

### 3.4. Determinants for Successful Implementation

EP mentioned several determinants for successful implementation, which we could categorize into four main themes: (a) time, (b) personal attitude, (c) mastery, and (d) complexity of the school environment. The themes are summarized with supporting quotes in Table 5. The biggest theme that emerged was that time is an important key for successfully implementing the SEd intervention. All EP reported to have too little time due to their high work-load and/or due to the intervention being more time-intensive than their standard way of counseling. The data suggest that the time-intensity the EP talked about was related to the preparation time they needed for each session and to the experience that the intervention included a great deal of writing during the sessions (e.g., filling in forms and diagrams together with the adolescent).

A second theme was personal attitude: for successful implementation of the SEd intervention in the counseling sessions, the method should be in line with the personal attitude of the EP towards supporting students. Some EP reported that the method fitted well with their own way of working because it fits well with their own attitude towards support and their previous experience (i.e., in solution-oriented coaching). These EP reported more positivity towards the implementation and also used the intervention with more adolescents.

A third theme was the mastery of the intervention. Most EP found that it took quite some time to learn and master the steps of the intervention and that even after the training, they still did not feel like they fully mastered the intervention and were able to adjust the steps based on the specific situation of the student.

The fourth theme was the complexity of the school environment. The organization and policies surrounding support/counseling differed per school. Differences were mentioned, for instance, in the amount of time that was available per session and per adolescent and the perspective of the school on the type of problems they are able to provide support for.

## 4. Discussion

With this study, we examined the feasibility of the SEd intervention by assessing the preliminary effectiveness, acceptability, and implementation from the perspective of the professionals as well as adolescents. First, participants in our small sample of adolescents reached or partly reached their goals and, on average, showed a slight decline in their level of psychosocial problems. Second, regarding acceptability, the EP and adolescents were positive about the intervention, especially about its step-wise approach and its structured way of counseling. The EP were also positive about the autonomy it induced in the adolescents. Finally, several factors limited the implementation: characteristics of the intervention, contextual factors, and personal beliefs of professionals.

The results regarding the preliminary effectiveness indicate a potential trend of reduction of psychosocial problems over time. This reduction is interesting since it is not the primary aim of the intervention, and it may be a side product of making problems more manageable [40]. Moreover, offering structure and stimulating autonomy may strengthen executive functioning skills and thus relieve the burden on their mental health and psychosocial functioning, as mental health and executive functioning are often related [41,42]. In addition, we found positive results in terms of goal attainment, which aligns with the main principles of the intervention. We found no changes in school functioning or general self-efficacy, which was contrary to our expectations. An explanation may be that our sample was too small to find such effects. In addition, in our sample, the adolescents with lower school functioning at the start of the intervention were more likely to drop out. This evidently deserves further attention in the intervention.

The SEd intervention as a new working method for secondary education was acceptable and useful for the professionals and adolescents, especially in terms of the structured, step-wise approach and the positive influence on the participation of adolescents. Often, adolescents in need of counseling have difficulties with executive functioning skills such as structuring [19]. Therefore, providing structure through the intervention can increase their competency in this area. Furthermore, the SEd intervention positively affected the autonomy and consequently the participation of adolescents in the counseling. Previous studies have shown that the participation of adolescents is an essential ingredient in the success of any type of care or support [43,44,45]. Adolescents may sometimes feel they have a limited role in the counseling process, so actively involving them can increase their willingness to participate in the decision making and their ownership of their problems and the solutions [46].

However, we found that despite good acceptability, the implementation was challenging due to certain characteristics of the intervention, the school context, and personal beliefs of the professionals. First, the EP made suggestions to improve the usability of the tools and manual, which have been updated. Amongst others, they had a need for a more concise manual, which also corresponds to their reported lack of time for counseling due to a high workload. Second, implementation was limited due to contextual factors, in particular, due to EP drop-out because of sick leave, changing jobs, and high workload. EP related their high workload partly to how the counseling is organized in the school. This corresponds with previous research in which researchers found that school organizational structures and changes, as well as the fit with school goals and policies, were among the most frequently found factors to limit implementation in schools [47,48]. Third, we found that personal beliefs of the professional played a role: more positive beliefs led to stronger involvement in the implementation of the intervention. This corresponds with previous findings that beliefs about the acceptability and efficacy of an intervention predict implementation commitment [49].

This study had several strengths and limitations. The main strengths of this study were its use of multiple informants and of a mixed-methods design. We included a heterogeneous sample of educational professionals from different schools. A main limitation is the high drop-out of EP during the quantitative part of the study, leading to a low inclusion of adolescents, which reduced the power of the study. This could have led to underestimation of effects, as those potentially benefitting the most were most likely to drop-out. Moreover, the small sample limited the analyses of the quantitative data and limited the generalizability of the results. In addition, we did not have a control group with which to compare the results, which limits our ability to draw definite conclusions from the quantitative data. Finally, we use the term “mental health problems” to refer to the participants with emotional or behavioral problems both with and without a diagnosis.

An important limitation was the drop-out of EP and the consequential low inclusion and drop-out of adolescents. An important question is whether this was due to the intervention itself or other factors. Based on the interview data, we conclude that the inclusion and drop-out problems were mainly due to the implementation issues related to the complex and often overloaded school environment. The intervention still seems positive and promising for those that participated but requires further study regarding effectiveness and wider implementation.

Our findings may have several implications for SEd and further research into this intervention. First, in regard to the intervention itself, suggestions of the EP are used to adapt the tools and manual of the intervention. Future research should investigate the effectiveness of this adapted version compared to a control group. Second, considering the factors related to the school environment that limited EPs’ implementation of the intervention in the current study, successful and sustainable implementation in the school environment requires a close collaboration and a strong collaborative process with schools. For instance, potential barriers due to the schools’ organization of support or their policies regarding support need to be thoroughly examined before the start of an implementation study to identify the necessary actions to create favorable conditions for implementation [50]. Third, several EP dropped out of the study due to factors such as sick leave, change of jobs, or lack of time. For future research, it is recommended to thoroughly screen professionals beforehand regarding their motivation and workload as well as to include rewards or some additional benefit for their participation. Fourth, we did not include opinions of the families of the adolescents. Considering that the family situation can be a part of the mental health problems of adolescents as well as a source of support, their role in the intervention deserves further study.

Our findings have several implications for practice. First, our findings suggest that SEd and its principles may offer a promising new route to overcome the barriers in educational success for adolescents with mental health problems. Our findings also highlight the barriers that EP still experience in implementation, mainly in regards to their workload and organizational issues. This implies that for future successful implementation of such an intervention in secondary school, attention should be focused on thorough preparation that involves the educators and the larger school organization. Creating a healthy school environment that supports adolescents with mental health problems thus starts with a school environment in which educational professionals are also adequately facilitated and supported. In such a structure, SEd and its principles may lead to major gains in students’ well-being and functioning.

## 5. Conclusions

Results on acceptability and preliminary effectiveness of the SEd intervention seem promising for those that participated in our study, suggesting that this intervention has the potential to help educational professionals as well as empower and support adolescents to participate in their education and take charge of their problems and the solutions. These results should be confirmed in a controlled intervention study.

## Figures and Tables

**Table 1:** The supported education intervention for adolescents in secondary school.

The supported education (SEd) intervention is an individualized instruction and support intervention, which aims to help people choose an education, obtain an education, and/or keep enrollment in an educational or vocational training program of their choice (Hofstra and Korevaar, 2016 [19]). In the current study, we only focus on the keep intervention. This intervention was developed to help (young) adults with mental health problems to remain in school by increasing their school success and satisfaction through the development of skills and the use of resources that are important in an educational setting. It is designed to help educational professionals (EP) who support (young) adults with mental health problems (e.g., emotional, behavioral, and/or social problems). The approach of SEd consists of:	
1. Goal setting:	The starting point is determined by investigating the problems from the perspective of the student as well as the school. The educational goal of the student is determined.
2. Examination of the problem and brainstorm the potential solutions:	Possible solutions to resolve the problems are examined together with the student as well as how they can reach their own educational goal.
3. Description and prioritizing the necessary critical skills and resources:	a. Functional assessment: The EP investigates, together with the student, which critical skills are needed to be successful and satisfied in a chosen educational setting. What skills does the student need to deal with the limitations of the educational setting? b. Resource assessment: The EP investigates, together with the student, which critical resources are needed to be successful and satisfied in the chosen educational setting.
4. Action plan:	The EP and student create an action plan together in order for the student to learn the necessary new skills and to organize the necessary critical resources. They define how these critical skills and resources are learned and organized (who, what, when, where).
5. Monitor and evaluate:	Monitor and evaluate whether the plan is executed and how the execution is going. Evaluate whether the plan needs adjustment.

Note: It is important that throughout the process, interpersonal skills such as listening, demonstrating understanding, and coaching/inspiring are used to connect and develop the relationship with the student.

**Table 2:** Characteristics of the Sample of Educational Professionals and Adolescents.

Educational Professionals (*n* = 8 ^a^)	
Gender female *n* (%)	7 (88%)
Age M(SD)	44 (12.2)
Adolescents (*n* = 18 ^a^)	
Gender female *n* (%)	10 (56%)
Age M(SD)	14 (1.7)
Grade *n* (%)	
Grade 1	2 (10%)
Grade 2	3 (17%)
Grade 3	6 (33%)
Grade 4	5 (28%)
Grade 5	1 (6%)
Grade 6	1 (6%)
Educational level *n* (%)	
Lower secondary	9 (50%)
Intermediate and Higher secondary	9 (50%)

^a^ Participants of the questionnaire.

**Table 3:** Outcomes of the SEd intervention (*n* = 7).

	T1	T2	T3
School functioning M(SD)	69 (8.6)	63 (11.0)	67 (13.2)
SDQ M(SD)	15 (4.0)	-	11 (3.5)
General self-efficacy M(SD)	30 (3.0)	-	30 (1.6)
Improved confidence (0–10) M (SD)	-	4.6	5.2
Improved understanding (0–10) M (SD)	-	6.9	6.5
Goal Attainment Scale (GAS)			
Obtained 1 or more goals *n* (%)	-	4 (57%)	5 (71%)
Partly obtained 1 or more goals *n* (%)	-	3 (43%)	2 (29%)

**Table 4:** Supporting quotes regarding the acceptability of the intervention.

Themes	Informant	Supporting Quotes
Acceptability		
Structure	EP	*“The supportive tools of the intervention were very useful. I can fill it in together with the adolescent, so she/he can see what we discuss. Sometimes if you just talk to an adolescent, it doesn’t really become tangible. But if you write it down together, it sticks.”* (EP4) *“There are a lot of papers in the manual, and a lot of the papers contain a lot of content. It would work better if everything is written down more concise. But it does also help, to have it as a reference. It would just be nicer if we also had everything in a more concise form, to work with.”* (EP3)
Autonomy	EP	*“They’ve become more independent. More goal-oriented. Because they talk more about what they want now. And how they can take charge, and understanding that they have to do it themselves.”* (EP1) *“He is able to pick up things on his own now, which I think is a very important benefit of the intervention, that he sees for himself: what can I do?”* (EP4)
Applicability	EP	*“It depends on the motivation of the adolescent, and the level of thinking, and what kind of home situation they are in, if they could do these steps and benefit from it. The adolescents that could benefit are the ones that are able to talk about and name their problems.”* (EP6) *“I don’t think there is a difference between different school levels. You have different conversations of course. But I explain everything on their level and I do think they all have an idea of what they want. When they enjoy school or what is important to them. They can all tell me that. And they all know that good grades are important.”* (EP3) *“I did not use this intervention with adolescents in lower secondary education because they often need short-term solutions. To correctly use this intervention, you need at least 6 to 8 sessions.”* (EP1)
Non-judgmental listening	Adolescent	*“Some teachers just get angry and the counselor just stays calm and listens. She is friendly and honest and respectful. I feel I can trust her.”* (A1)
Shared decision making	Adolescent	*“We talked about solutions for my concentration problems, and looked at several options together. Then we tried two solutions, the counselor suggested using a stress ball and mine was listening to my mp3 player when I work on assignments in the classroom.”* (A2)
Structure	Adolescent	*“We sort of kept replaying the story: what actually happened, when was the exact point at which things went wrong, when I got angry. And then we talked about what I could do differently next time. I sort of have a strategy now. I am able to stay calm and think about the situation. Think about it step by step. And how to solve it.”* (A4)
Visualization	Adolescent	*“We wrote down on the whiteboard what I need and what I could do [...] That really makes it clear. Then I can also see what I said.”* (A1)

**Table 5:** Supporting quotes regarding implementation of the intervention.

Themes	Informant	Supporting Quotes
Implementation		
Time	EP	*“Educational professionals are often caught in the daily issues. Something new happens every day, and next to that, as a counselor you are caught in between various parties, internally and externally. And there is a lot more work involved if you want to do it right. I think that it is too burdensome for the time most professionals have.”* (EP1) *“The intervention becomes easier the more you work with it. But right now, you have to read up on the method every time, which costs time, reporting costs time, it just costs a lot of time. And we do not have that.”* (EP7)
Personal attitude	EP	*“I think EP are just used to taking care of things for adolescents. The intervention is more leaning back and I think they need to practice that. To sit back and let the adolescent do more themselves. That’s what I see happening with my colleagues.”* (EP1) *“I am convinced that it is so valuable if you let the adolescent think for themselves more, about their problems and the potential solutions. The adolescent being the central point in counseling is very important and I could not work differently.”* (EP3)
Mastery	EP	*“Perhaps if you are working with this method for a longer time and when it has become more of a routine, when you don’t have to prepare so much, then you can easily use this intervention. But I don’t feel like I’m there yet.”* (EP4) *“I tried working with the whole method, but I did skip some steps sometimes due to practical reasons. Sometimes I thought I was just repeating myself or in other cases it did not seem useful for that person. In that sense it was too extensive for me, the method. And then I chose to be flexible, to adapt it to the adolescent, and maybe skip a step.”* (EP7)
Complexity of school environment	EP	*“It is policy in our school that counseling is 7 weeks, and this intervention barely fits in those weeks. If it helped, okay, then we stop. Someone else must also get their turn. Or in some cases you continue, but only when you notice a positive effect, but haven’t seen much concrete results yet.”* (EP2) *“With these kind of support interventions, not just this intervention, but in general, you are very dependent on the organizational structure of the school. It is a choice of our school. We just want the kids to get support as soon and as fast as possible. And that is at the expense of this intervention, which is a more extensive method.”* (EP7)

## Data Availability

Data are available upon request.

## Appendix A

**Table A1:** Learning goals of the training.

	Learning Goals:
Meeting 1	Understanding the mission and underlying framework of the intervention.
	Understand and practice step 1 of the intervention: - What are the adolescents’ problems, and whose problem is it? - What are the adolescents’ goals for the counselling?
	Understand and practice step 2: - Determine barriers adolescent faces in school (i.e., is there a mismatch between demands and needs?) - Determine which skills and resources the adolescent needs to deal with the barriers.
Meeting 2	Understand and practice step 3: - Which skills does the adolescent need to learn or learn to apply? - Which resources does the adolescent need to realize or organize? - Prioritize the necessary skills and resource.
Meeting 3	Understand and practice step 4: Developing an Individual Development Plan.
	Understand and practice step 5: Execute, monitor, and evaluate the Individual Development Plan (is it executed, how do you monitor, and does the plan need to be adjusted).
Meetings 4, 5, 6	Discuss case studies, questions, and problems of the participants.
	Practice (parts of) the intervention with other participants.
	Reflect on and evaluate the intervention and the training.
For more information on the theoretical background of the intervention and the training, see Hofstra and Korevaar, 2016 [19])	
